# IL-27-Induced Type 1 Regulatory T-Cells Produce Oxysterols that Constrain IL-10 Production

**DOI:** 10.3389/fimmu.2017.01184

**Published:** 2017-09-25

**Authors:** Solenne Vigne, Fanny Chalmin, Donovan Duc, Aurélie S. Clottu, Lionel Apetoh, Jean-Marc A. Lobaccaro, Isabelle Christen, Juan Zhang, Caroline Pot

**Affiliations:** ^1^Laboratories of Neuroimmunology, Division of Neurology and Neuroscience Research Center, Department of Clinical Neurosciences, Lausanne University Hospital, Lausanne, Switzerland; ^2^Department of Pathology and Immunology, University of Geneva, Geneva, Switzerland; ^3^Faculté de Médecine, University of Bourgogne, INSERM U866, Centre Georges François Leclerc, Dijon, France; ^4^GReD, Université Clermont Auvergne, CNRS, INSERM, CRNH Auvergne, Clermont-Ferrand, France; ^5^Analytical Sciences and Imaging, Novartis Institutes for BioMedical Research, Basel, Switzerland

**Keywords:** immunometabolism, CD4^+^ T cells, type 1 regulatory T cells, autoimmunity, oxysterols, cholesterol 25-hydroxylase, Epstein–Barr virus-induced G-protein coupled receptor 2 (EBI2), nuclear hormone liver X receptor

## Abstract

The behaviors of lymphocytes, including CD4^+^ T helper cells, are controlled on many levels by internal metabolic properties. Lipid metabolites have recently been ascribed a novel function as immune response modulators and perturbation of steroids pathways modulates inflammation and potentially promotes a variety of diseases. However, the impact of lipid metabolism on autoimmune disease development and lymphocyte biology is still largely unraveled. In this line, oxysterols, oxidized forms of cholesterol, have pleiotropic roles on the immune response aside from their involvements in lipid metabolism. The oxysterols 25-hydroxycholesterol (25-OHC) and 7α,25-dihydroxycholesterol (7α,25-OHC) regulate antiviral immunity and immune cell chemotaxis. However, their physiological effects on adaptive immune response in particular on various subset CD4^+^ T lymphocytes are largely unknown. Here, we assessed oxysterol levels in subset of CD4^+^ T cells and demonstrated that 25-OHC and transcript levels of its synthesizing enzyme, cholesterol 25-hydroxylase, were specifically increased in IL-27-induced type 1 regulatory T (T_R_1) cells. We further showed that 25-OHC acts as a negative regulator of T_R_1 cells in particular of IL-10 secretion *via* liver X receptor signaling. Not only do these findings unravel molecular mechanisms accounting for IL-27 signaling but also they highlight oxysterols as pro-inflammatory mediators that dampens regulatory T cell responses and thus unleash a pro-inflammatory response.

## Introduction

Oxysterols, oxidized forms of cholesterol, are essential precursors for bile acid and steroid biosynthesis. Apart from their basic metabolic properties, they have recently been ascribed with immunomodulatory functions. The enzyme cholesterol 25-hydroxylase (Ch25h) is the rate-limiting step to synthetize both 25-hydroxycholesterol (25-OHC) and 7α,25-dihydroxycholesterol (7α,25-OHC) from cholesterol. Both oxysterols modulate the immune response, 25-OHC controls viral infection in macrophages (1) and 7α,25-OH promotes macrophage and B cell trafficking within lymphoid structures (2). We showed that 7α,25-OHC promotes memory CD4^+^ T cell migration to the target inflammatory organs during autoimmunity (3, 4). While authors have proposed oxysterols as pro-inflammatory mediators, others have submitted 25-OHC as an anti-inflammatory intervener (5). Those contradictory results open the debate on the biological activities of oxysterols during the immune response. Furthermore, while the roles of oxysterols during innate immune response have been well studied in macrophages, their tasks during adaptive immune response remain largely unknown.

Adaptive immune homeostasis relies in part on orchestrated interactions among subsets of T cells with effector or regulatory functions. CD4^+^ regulatory T cell subsets include naturally occurring CD4^+^CD25^+^ Treg cells (nTregs), which can be defined by their expression of the forkhead-box transcription factor Foxp3, as well as peripherally induced type 1 regulatory T (T_R_1) cells that produce IL-10. The cytokine IL-27, mainly produced by antigen-presenting cells, promotes T_R_1 cell development. While initial animal studies suggested that IL-27 supported pro-inflammatory responses, the anti-inflammatory properties of IL-27 were exemplified in mouse models, where IL-27 injections reduced disease severity of experimental autoimmune encephalomyelitis (6–8). In addition, IL-27R-deficient mice show enhanced pro-inflammatory CD4^+^ T cell response and enhanced autoimmunity susceptibility (9, 10) and die following exposure to parasitic and bacterial infections due to severe immunopathology (11). IL-27 downmodulates the immune responses through production of the immunosuppressive cytokines IL-10 (12) and IFN-γ (7) and by inhibiting pro-inflammatory cytokine, including IL-17, production (6). Interestingly, the oxysterol 7β,27-dihydroxycholesterol has been identified as an agonist for RORγt, a crucial transcription factor for IL-17-producing CD4^+^ T cells (T_H_17 cells) and, thus, as a pro-inflammatory mediator (13). Those results suggest that oxysterols could act as fine tuners of the immune response.

Here, we show that the oxysterol 25-OHC is specifically induced by IL-27 *via* the signal transducer and activator of transcription factor 1 (Stat1) and interferon regulatory factor 1 (IRF1) signaling during CD4^+^ T cell differentiation. 25-OHC further acts as a negative regulator on IL-10 production by lowering B-lymphocyte-induced maturation protein 1 (Blimp1) expression that contributes to IL-10 secretion by CD4^+^ T cells (14). 25-OHC dampens anti-inflammatory cytokine production *via* the nuclear hormone liver X receptors (LXR) signaling and further promotes intracellular cholesterol accumulation, a process recognized to drive inflammation (15). Those results strengthen the pro-inflammatory role of 25-OHC during adaptive immune response by limiting the generation of IL-27-induced regulatory T_R_1 cells both *in vitro* and *in vivo*.

## Materials and Methods

### Animals

*Ch25h*^−/−^ mice were purchased from Jackson Laboratory. Stat1^−/−^ mice on C57BL/6 background were a kind gift from Professors M. Mueller and D. Merkler (16), *Irf1*^−/−^ by L. Apetoh (17), and *Lxr*αβ^−/−^ by D. J. Mangelsdorf (18). Mice, on C57BL/6 background, were housed under specific pathogen-free conditions at Lausanne University Hospital. All experiments were undertaken in accordance with guidelines from the Cantonal Veterinary Services of states Vaud and Geneva.

### *In Vitro* T Cell Differentiation

Spleen and inguinal lymph nodes were obtained from 6- to 10-week-old mice and then mashed on a 70-µm mesh together with culture media to obtained single cell suspension. After erythrocyte lysis, naive CD4^+^ T cells were purified by negative selection using immunomagnetic beads (Naive CD4^+^T cell Isolation Kit, Miltenyi Biotec) and stimulated for 3 days or for the indicated time on plate-bound antibodies against CD3 (145-2C11, 1 µg/ml) and CD28 (PV-1, 1 µg/ml) without cytokines (T_H_0) or with mouse IL-27 [50 ng/ml (T_R_1); IL-12 (10 ng/ml) and anti-mouse IL-4 (11B11; 20 µg/ml) (T_H_1); IL-4 (20 ng/ml) and anti-mouse IFNy (XMG1.2; 20 µg/ml) (T_H_2); human TGF-β1 (2 ng/ml) (iTregs); TGF-β1 and IL-6 (20 ng/ml) (T_H_17)]. Cytokines were purchased from eBioscience, 25-OHC from Avanti Polar Lipis Inc., anti-CD3/CD28 monoclonal antibodies (mAbs) from BioXcell and LXR agonists T0901317 and GW3965 from Sigma.

### Oxysterol Extraction and Analysis using Ultra-High Performance Liquid Chromatography–Tandem Mass-Spectrometry (UHPLC–MS/MS)

Cell pellets (10^6^ cells) re-suspended in water containing a mixture of deuterated internal standard compounds and 200 µM Butylated hydroxytoluene were lysed using a Precellys^®^24 (Bertin Technologies) before extraction. EtOH (ninefold in volume) was added in six steps to allow a slow protein precipitation under 4°C. One milliliter cell culture medium was used to extract oxysterols using the same slow protein precipitation method. Extracts were dried down and concentrated 10 times prior to injection into UHPLC-MS/MS. The oxysterols analysis was carried out on a Nexera UHPLC system (Shimadzu, Kyoto, Japan) coupled to a QTrap^®^6500 (ABSciex, Framingham, MA, USA) mass spectrometer. The UHPLC-MS/MS method was as previously described (19).

### Cytokine Measurements

Supernatants were collected after 48 h of culture and secreted cytokines measured by ELISA (eBioscience).

### Flow Cytometry

Cells, preincubated with mAb 2.4G2 (anti-CD16/32) to block Fc receptors were labeled with CD4 AlexaFluor 700 (GK 1.5, eBioscience). Cytokine-detection was performed by intracellular cytokine staining with anti-IFN-γ Alexa Fluor 488 (XMG1.2, eBiosciences) and anti-IL-10 BV421 (JE55-16E3, Biolegend). Cells (1.5 × 10^5^ cells/well) were stimulated at 37°C with 10 ng/ml phorbol myristate acetate (PMA, Sigma), 1 µg/ml ionomycin (Sigma) for 4 h, and 5 µg/ml Brefeldin (Sigma) for 2 h and permeabilized using Foxp3/Transcription Factor Staining Buffer Set (eBiosciences). Data were acquired using a LSR II cytometer (BD Biosciences).

### CFSE Labeling

Naive CD4^+^ T cells, suspended in 5-µM CFSE staining buffer (Molecular Probes) and incubated at 37°C for 10 min, were cultured as described above. After 5 days of culture, cell division was determined by measuring CFSE fluorescence in total cells. Cell viability was determined by measuring Fixable viability Stain 620 (FVS620, BD Bioscience). Data were analyzed using the FlowJo V10 software.

### Protein Isolation and Analysis

Total cell lysates were prepared in RIPA buffer (50 mM Tris–HCl pH 7.4, 2 mM EDTA pH8, 150 mM NaCl, 0.5% Na-deoxycholate, 0.1%SDS, 1% Non-idet P40) supplemented with protease inhibitors (Mini protease inhibitor, Roche). Samples were separated on a 8% SDS-polyacrylamide gel and transferred to nitrocellulose. Rat anti-mouse Blimp1 (5E7, Santa Cruz) at 1/200 dilution, rabbit anti-mouse β-Actin (N-21, Santa Cruz) at 1/600, Goat anti-rat or rabbit IgG-HRP 1/10000 were used and visualized by chemiluminescence (ECL, Amersham Pharmacia Biotech); relative density of Blimp1 expression analyzed using the ImageJ software.

### Quantitative Real-time PCR (RT-PCR)

RNA was extracted with Tryzol (Invitrogen Life Technologies), cDNA synthesized with random hexamers and Superscript II reverse transcriptase (Invitrogen Life Technologies) used as template for RT-PCR (Applied Biosystems^®^ StepOne plus) with SYBR green Supermix (KAPA SYBR^®^ FAST Universal, Labgene). Gene expressions were assessed with specific primers as follows: Ch25h (Fw CCAGCTCCTAAGTCACGTC Rev CACGTCGAAGAAGGTCAG), Cyp7B1 (Fw TTCCTCCACTCATACACAATG Rev CGTGCTTTTCTTCTTACCATG), HSD3B7 (Fw AAGAGGCCAGCAATACCCAG Rev ACCATCCACAAAGTCAACG), Blimp1 (Fw GGAGGATCTGACCCGAAT Rev TCCTCAAGACGGTCTGCA), AhR (Fw CTCCTTCTTGCAAATCCTGC Rev GGCCAAGAGCTTCTTTGATG), c-maf (Fw GGCCATGGAATATGTTAATGACTTC Rev CCGCACTGGCTGATGATG), IRF1 (Fw AGGCATCCTTGTTGATGTCC Rev AATTCCAACCAAATCCCAGG), LXR-β (Fw TTTGCTTTTCGCTCAGCAAGC Rev GGAGGCGAGAGTTGCCTCTG), SREBF1 (Fw GGGGAACTTTTCCTTAACGTGG Rev CGGGAAGTCACTGTCTTGGT), ABCA1 (Fw AGCACCGTGTCTTGTCTGAA Rev CATCGATGGTCAGCGTGTCA) and β-actin (Fw CCTGTATGCCTCTGGTCGTA Rev CCATCTCCTGCTCGAAGTCT). Values obtained with the SDS 2.2 software (Applied Biosystems) and gene expression calculated using the comparative method (2^−ΔCt^) for relative quantification by normalization to β*-actin* gene expression.

### *In Vivo* Treatment with Anti-CD3

*Ch25h*^−/−^ and wild-type mice were treated with 20 µg of anti-CD3 (clone 2C11) or PBS i.p. every 3 days for a total of four times. Mice were sacrificed 4 h after the last treatment, single cell suspensions were prepared from mesenteric lymph nodes (MLNs).

### Statistical Analysis

Statistical analysis was performed using Prism software (Graph Pad software, La Jolla, CA, USA). Evaluations were performed with the unpaired Student’s *t* test or with two-way ANOVA as appropriate. Two-tailed *p*-values < 0.05 were considered significant.

## Results

### IL-27 Induces Ch25h Expression and Production of 25-OHC

**Figure 1:**
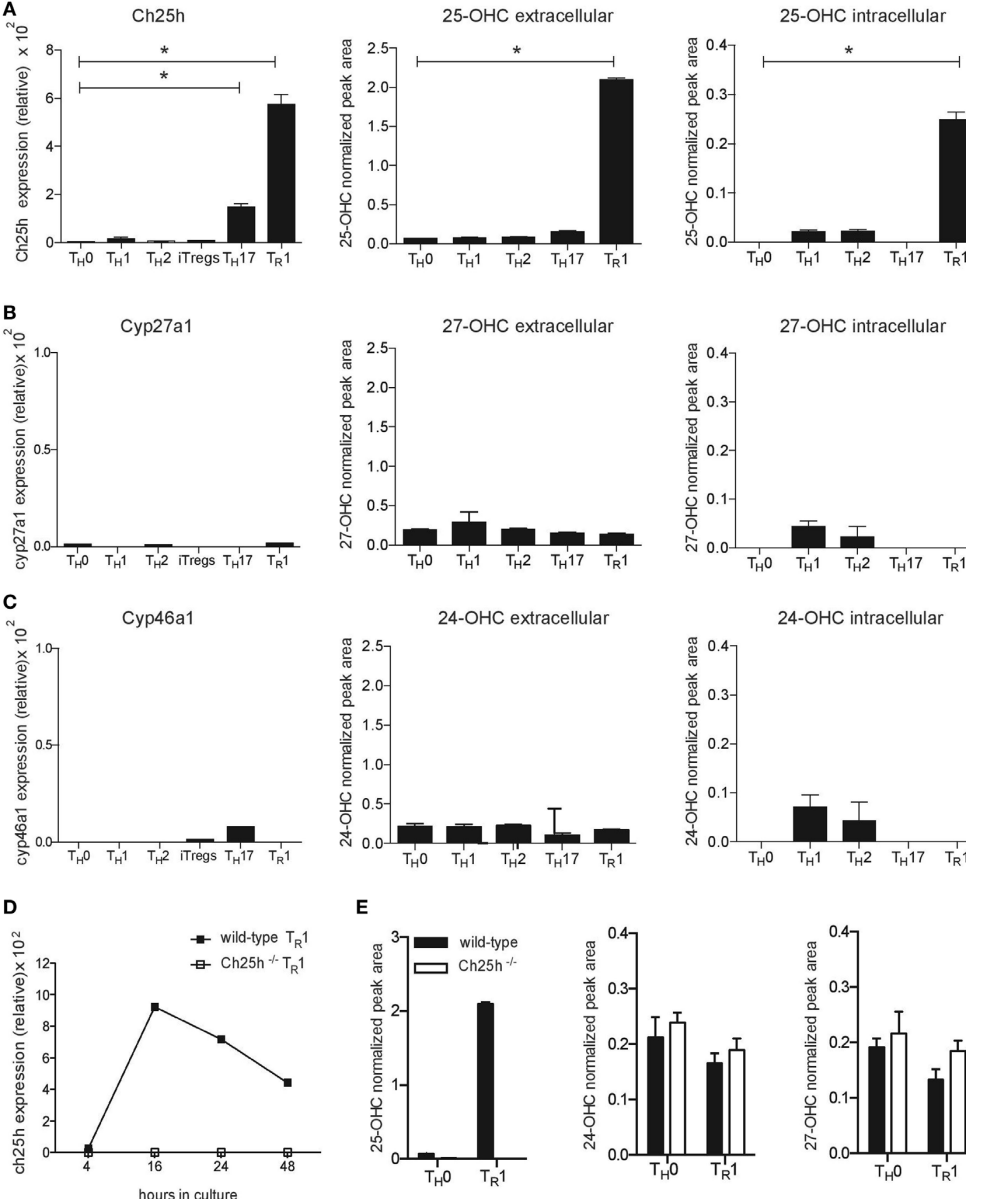
IL-27 specifically induces cholesterol 25-hydroxylase (Ch25h) expression and 25-OHC production in CD4^+^ T lymphocytes. Naive CD4^+^CD62L^hi^CD25^−^ T cells obtained from wild-type mice were differentiated into T_H_0, T_H_1, T_H_2, T_H_17, iTregs, and T_R_1 cells in the presence of anti-CD3 and anti-CD28 antibodies. RNA isolated from the CD4^+^ T cells after 24 h of culture was subjected to real-time PCR (RT-PCR) relative to the expression of mRNA encoding β-actin (2^−ΔCT^ × 100,000) to examine oxysterol-converting enzyme expression, while oxysterol levels were assessed by LC–MS/MS in supernatants (extracellular) and in cell pellets (intracellular) after 3 days of culture. **(A)** Ch25h expression and 25-OHC production **(B)** Cyp27a1 expression and 27-OHC production. **(C)** Cyp46a1 expression and 24-OHC production. **(D)** RNA isolated at different time points of culture following activation with IL-27 from naive CD4^+^ T cells obtained from wild-type mice (closed squares) or Ch25h^−/−^ mice (open squares), was subjected to RT-PCR to examine Ch25h expression. **(E)** Extracellular oxysterols levels measured by LC–MS/MS after 3 days of culture from wild-type mice or Ch25h^−/−^ naïve CD4^+^ T cells differentiated in the presence (T_R_1) or absence (T_H_0) of IL-27. Data are shown from two or three independent experiments (*p < 0.05).

While oxysterols are implicated in immune responses, their levels in T lymphocytes have not been assessed. We investigated the expression of oxysterol-converting enzymes on different subsets of T helper CD4^+^ T cells differentiated *in vitro* into T_H_0, T_H_1, T_H_2, T_H_17, Foxp3iTregs, or T_R_1 cells. By quantitative RT-PCR analysis, we observed that Ch25h was highly expressed in IL-27-induced T_R_1 cells, compared to cells activated in the absence of differentiating cytokines (T_H_0) (Figure 1A, left panel). Cholesterol is converted by Ch25h into 25-OHC. We, therefore, applied UHPLC-MS/MS to analyze the extra- and intracellular oxysterol levels. Consistent with Ch25h-increased expression, 25-OHC production was specifically induced by IL-27 and detected at high levels in T_R_1 cells. Low level of Ch25h expression was observed in T_H_17 cells, but 25-OHC production was not increased in T_H_17 compared to T_H_0 subset (Figure 1A, right panels). 25-OHC can be further metabolized into 7α,25-OHC. However, 7α,25-OHC could not be detected in any subset of T cells. We further examined other oxysterol-converting enzyme expressions and observed marginal expressions of the oxysterol-converting enzymes Cyp27a1 (Figure 1B) and Cyp46a1 (Figure 1C) in all T cell subsets without any specific induction by differentiating cytokines. The oxysterols 27-OHC downstream Cyp27a1 (Figure 1B) and 24-OHC downstream Cyp46a1 (Figure 1C) were detected in all cell types at very low levels and their productions not affected by any cytokine combinations.

25-OHC can also be formed from cholesterol through autoxidation (20) or by alternate pathways (21). We, therefore, differentiated T_R_1 cells from both wild-type and Ch25h^−/−^ CD4^+^ T cells to assess Ch25h-independent production of 25-OHC. IL-27 induced a significantly increased expression of Ch25h in wild-type CD4^+^ T cells starting after 16 h until 48 h of culture compared to Ch25h^−/−^ cells, where no Ch25h expression was detected (Figure 1D). IL-27 did not induce 25-OHC production in the absence of Ch25h and no compensatory increase of 24-OHC or 27-OHC production was observed in Ch25h^−/−^ T_R_1 cells (Figure 1E).

### IL-27 Induces Ch25h in a Stat1- and Irf-1-Dependent Manner

**Figure 2:**
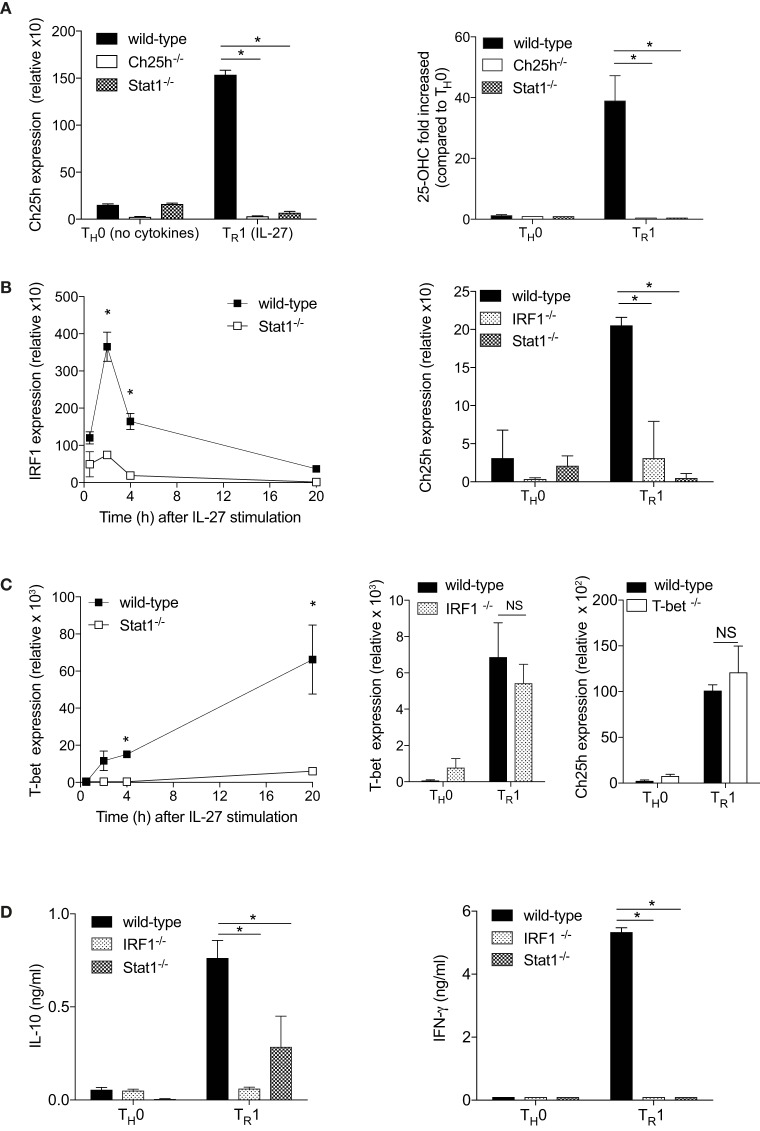
The transcription factors Stat1 and interferon regulatory factor 1 (IRF1) are mandatory for cholesterol 25-hydroxylase (Ch25h) induction by IL-27. Naive CD4^+^ T cells obtained from wild-type, Ch25h^−/−^, Stat1^−/−^, and IRF1^−/−^ mice were differentiated without cytokines (T_H_0) or with IL-27 (T_R_1) as indicated. **(A)** Ch25h expression was assessed by real-time PCR (RT-PCR) relative to β-actin expression after 24 h in culture (left panel) and extracellular 25-OHC levels measured by LC-MS/MS (right panel). **(B)** IRF1 and Ch25h expression levels were assessed by RT-PCR at the indicated time points following activation (left panel) or after 24 h of culture (right panel). **(C)** T-bet and Ch25h expression levels were assessed by RT-PCR at indicated time points (left panel) or after 24 h of culture *p < 0.05. **(D)** IL-10 (left panel) and IFN-γ (right panel) secretions were measured by ELISA in the supernatants of T cells cultured for 48 h. Data are shown from one out of three independent experiments (*p < 0.05).

IL-27 signals through Stat1 and Stat3 (22, 23). Because Stat1 induces Ch25h expression in macrophages (1), we tested the ability of IL-27 to activate Ch25h expression in Stat1^−/−^ cells. Genetic elimination of Stat1 resulted in the marked loss ability of IL-27 to induce Ch25h (Figure 2A, left panel). Moreover, the ability of IL-27 to induce 25-OHC in CD4^+^ T cells was abrogated in the absence of Stat1 (Figure 2A, right panel).

Interferon regulatory factor 1 is a main transcription factor downstream Stat1 (17, 24) that has been proposed to drive Ch25h expression during viral infection (25). IRF1 is induced by IL-27 in a Stat1-dependent manner, with an early peak expression after 2 h of culture (Figure 2B, left panel). We further asked whether Ch25h expression was dependent on IRF1 and tested the ability of IL27 to activate Ch25h mRNA levels in IRF1^−/−^ CD4^+^ T cells. Similarly to Stat1^−/−^ T cells, IL-27 was not able to induce Ch25h in the absence of IRF1 (Figure 2B, right panel). T-bet, another transcription factor downstream of Stat1, is induced by IL-27 with high expression 20 h after culture initiation (Figure 2C, left panel). However, T-bet expression was independent of IRF1 as IRF1^−/−^ T cells expressed T-bet at similar levels as wild-type T cells (Figure 2C, middle panel), suggesting that Ch25h expression is not downstream of T-bet. Indeed IL-27 could induce Ch25h expression in the absence of T-bet (Figure 2C, right panel). We, therefore, propose that the transcription factor IRF1, but not T-bet, is mandatory for IL-27-induced Ch25h expression.

T_R_1 cells are characterized by their secretion of IL-10 and IFN-γ (22). We observed that IRF1 and Stat1 are important to maintain both IL-10 and IFN-γ expression, as in the absence of each individual transcription factors, cytokine expressions were significantly reduced (Figure 2D).

### Ch25h-Deficient T_R_1 Cells Depict Higher IL-10 Production both *In Vitro* and *In Vivo*

**Figure 3:**
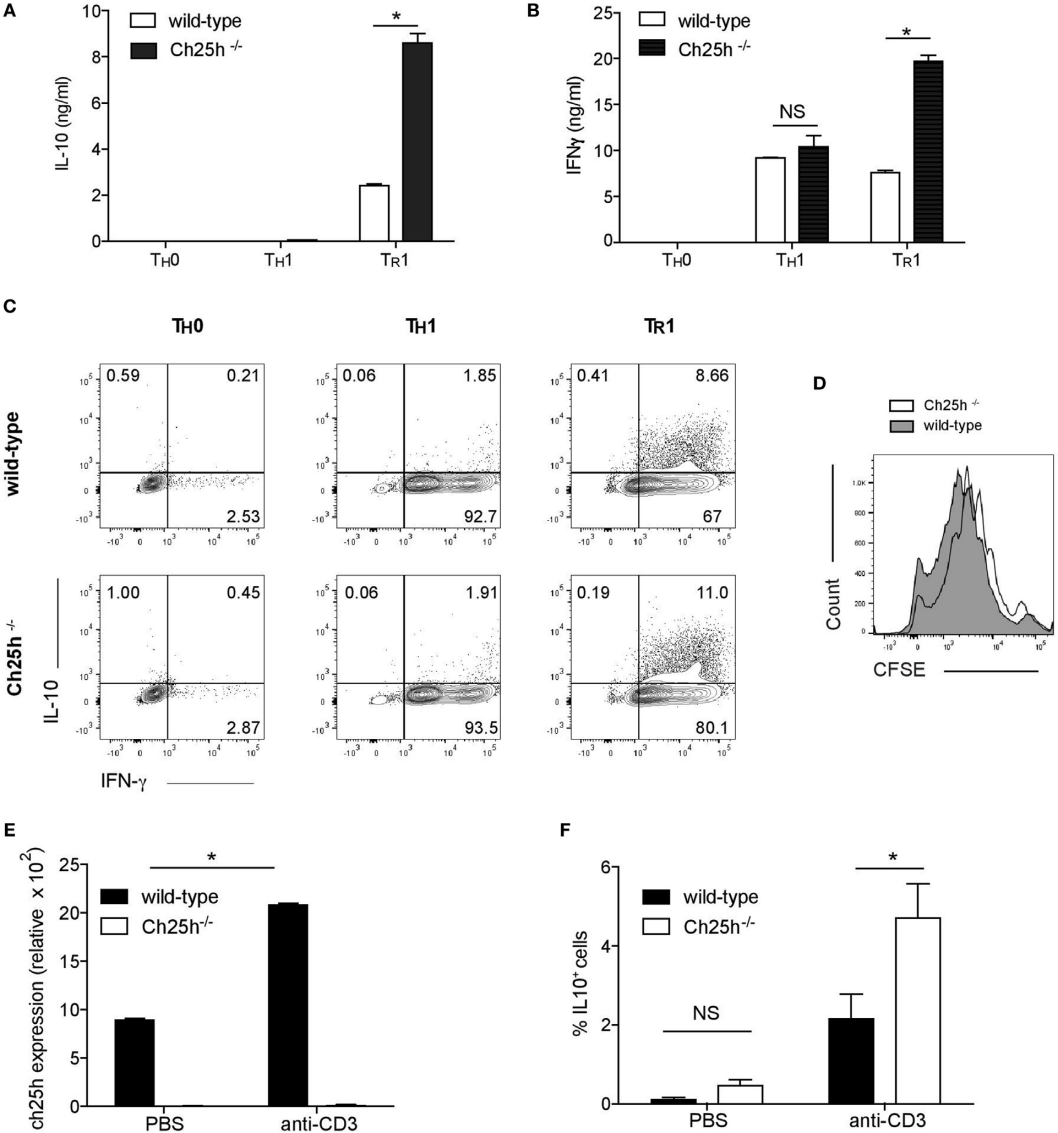
Endogenous 25-OHC negatively regulates IL-10 production in T_R_1 cells *in vitro* and *in vivo*. Naive CD4^+^ T cells obtained from wild-type or Ch25h^−/−^ mice as indicated were differentiated into T_H_0, T_H_1, and T_R_1 cells for 48 h. **(A,B)** IL-10 and IFN-γ cytokine productions in culture supernatants were assessed by ELISA. Data are shown from one of three independent experiments with similar results. Error bars represent the SD of triplicates in the same experiment (*p < 0.05). **(C)** Flow cytometric analysis of intracellular staining of IL-10 and IFN-γ of T_H_0, T_R_1 cells, and T_H_1 cells. **(D)** Proliferative responses were assessed by CFSE incorporation in T_R_1 cells, representative of one of three independent experiments with similar results. **(E)** Wild-type or Ch25h^−/−^ mice were injected i.p. with 20 µg of antibodies to CD3 or PBS once every 3 days, for a total of three times. 4 h after the last injection, mice were sacrificed. CD4^+^ T cells from mesenteric lymph nodes (MLN) were then FACS sorted, RNA was then isolated and subjected to real-time PCR to examine cholesterol 25-hydroxylase (Ch25h) expression. **(F)** Frequency of IL-10^+^ CD4^+^ T cells from anti-CD3 or PBS treated mice was analyzed by flow cytometry in MLN (mean + SD of three mice). Data are shown from one out of two experiments (*p < 0.05).

The strong expression of Ch25h induced by IL-27 prompted us to investigate the role of 25-OHC during T_R_1 cell differentiation. Naive CD4^+^ T cells from wild-type or Ch25h^−/−^ mice were differentiated *in vitro* with IL-27 (T_R_1), without any cytokines (TH0) or with IL-12 and anti-IL-4 to generate T_H_1 as a control as they also express IFN-γ. The secretion of IL-10 (Figure 3A) and IFN-γ (Figure 3B) were notably enhanced in T_R_1 cells derived from Ch25h^−/−^ cells (white bars) compared to wild-type cells (black bars). IFN-γ was not enhanced in T_H_0 nor in T_H_1 cells. Furthermore, the frequency of cell expressing IL-10 and IFN-γ was increased in Ch25h^−/−^ compared to wild-type T_R_1 cells while they were not enhanced neither in T_H_0 nor in T_H_1 cells (Figure 3C). Oxysterols interfere with different cell type proliferation, including cancer cells (26). However, CFSE staining showed similar proliferation rates between Ch25h^−/−^ and wild-type Tr1 cells (Figure 3D).

To further address the *in vivo* relevance of Ch25h in inducing IL-27-driven T_R_1 cells and the potential effect on regulating autoimmunity and tissue inflammation, we conducted repeated *in vivo* treatments with anti-CD3 to induce IL-10^+^ regulatory T cells (27), that have been shown to be IL-27 dependent (28). We, thus, repeatedly administered anti-CD3 or PBS to C57Bl6 wild-type mice and assessed Ch25h expression in MLNs 4 h after the last injection. In line with our *in vitro* findings, Ch25h was significantly induced in wild-type but not Ch25h^−/−^ CD4^+^ T cells (Figure 3E). Since IL-10 is produced by T_H_17 cells (29), Foxp3^+^ Tregs (30), and T_R_1 cells, we further analyzed the production of IL-10 by Foxp3*^−^* IL-17*^−^* CD4^+^ CD3^+^ TCRαβ^+^ T cells as previously published (28). Administration of anti-CD3 to wild-type mice resulted in a significant induction of IL-10^+^ T cells in the MLNs that were significantly increased in Ch25h^−/−^ mice (Figure 3F). Ch25h, thus, inhibits IL-10^+^ T cell generation both *in vitro* and *in vivo*.

### 25-OHC Impairs IL-10 Expression from IL-27-Induced T_R_1 Cells

**Figure 4:**
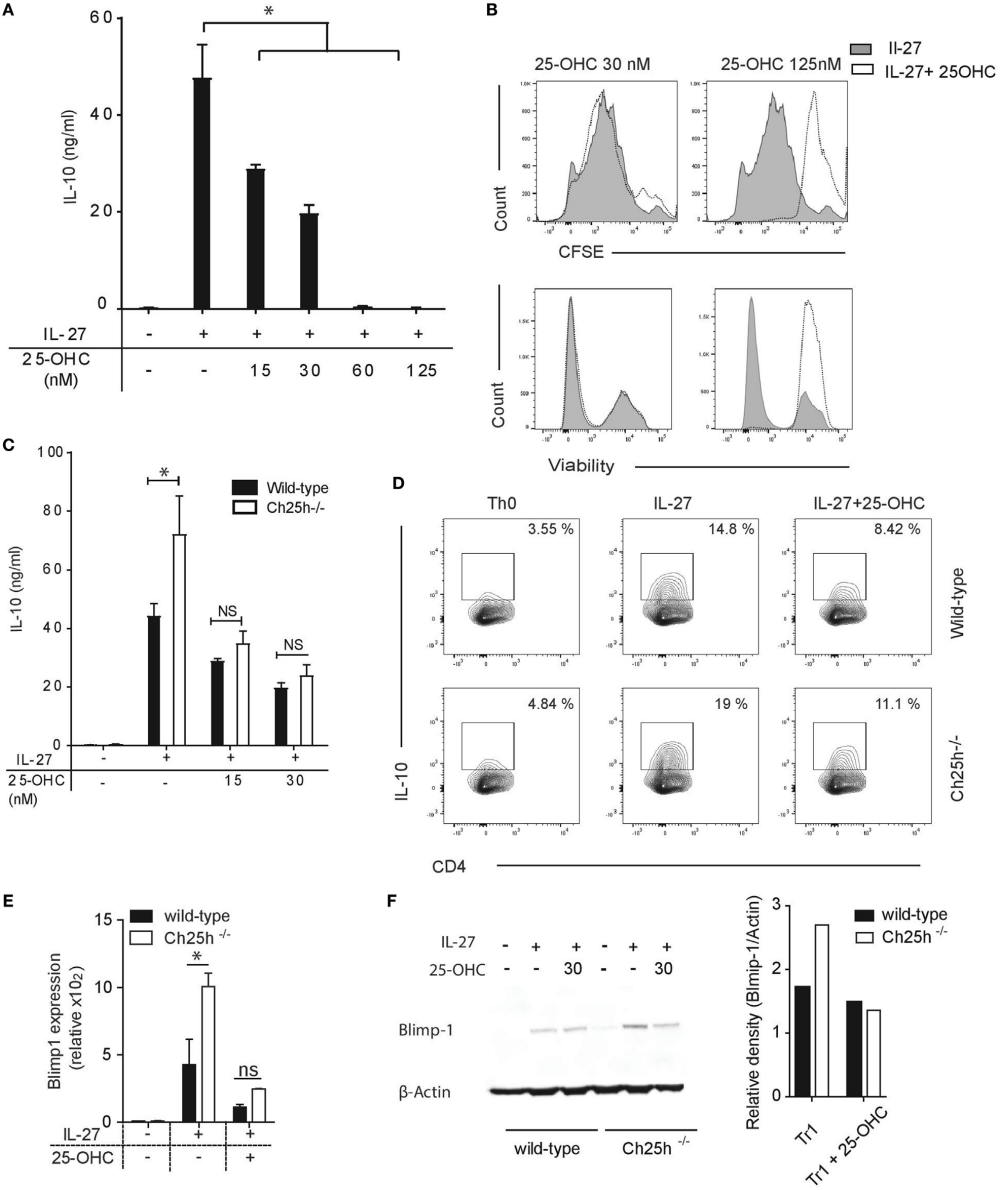
Exogenous 25-OHC inhibits T_R_1 cell differentiation. Wild-type or Ch25h^−/−^ naive cells were differentiated with IL-27 in the presence of 25-OHC at the indicated concentration (or with 30 nM if not indicated) **(A)** IL-10 cytokine production in culture supernatants assessed by ELISA analysis. **(B)** Histogram profiles of CFSE (above) and fixable viability-labeled cells, with highly stained cells corresponding to dead cells (below). **(C)** IL-10 cytokine production assessed by ELISA analysis and **(D)** IL-10-expressing CD4^+^ T cells detected by intracellular staining and quantified by flow cytometry. Data shown are representative of one of three independent experiments with similar results (*p < 0.05). B-lymphocyte-induced maturation protein 1 (Blimp1) expression was evaluated by **(E)** quantitative real-time PCR relative to β-actin (*p* < 0.05). **(F)** Western blot on whole cell lysates. Relative density values of Blimp1 were calculated using ImageJ software. Data are shown from one of two independent experiments with similar results (*p < 0.05).

We further asked whether exogenous 25-OHC influences IL-10 production. Addition of 25-OHC during T_R_1 cell differentiation decreased IL-10 secretion in a dose-dependent manner (Figure 4A). 25-OHC did not inhibit T_R_1 cell proliferation assessed with CFSE, nor impacted Tr1 cell viability at concentration of 30 nM or lower (Figure 4B). At higher doses, in addition to the effects on IL-10 secretion, proliferation was inhibited and cell viability decreased (Figure 4B). We thus pursued our experiments with concentrations of 25-OHC that solely impacted cytokine production. We further questioned whether the unique addition of 25-OHC would compensate for the IL-10 phenotype noted in Ch25h^−/−^ T_R_1 cell. The sole addition of 25-OHC (at 15 and 30 nM) dampened both IL-10 secretion (Figure 4C) and IL-10 frequency (Figure 4D) in Ch25h^−/−^ T cells reversing to similar IL-10 level of wild-type T_R_1 cell.

We then assessed if oxysterols impact the expression levels of transcription factors involved in IL-10 production. Ahr, c-maf, and Blimp1 control IL-10 expression during T_R_1 cell differentiation (14, 28, 31). While Ahr and c-maf expressions were not increased in Ch25h^−/−^ T_R_1 cells (Figure S1A in Supplementary Material), Blimp1 expression level was increased in Ch25h^−/−^ T_R_1 cells and downregulated by 25-OHC both at the mRNA (Figure 4E) and protein levels (Figure 4F). We further observed that Blimp1 expression was dependent on IRF1 and Stat1 signaling (Figure S1B in Supplementary Material), both of which showed to be critical for Ch25h expression. Those results suggest that 25-OHC negatively regulates IL-10 by dampening Blimp1 expression.

Altogether, these findings suggest that Ch25h-signaling pathway negatively regulates IL-10 expression in IL-27-induced T_R_1 cells.

### Oxysterols Inhibit IL-10 Secretion in an LXR-Dependent Manner in T_R_1 Cells

**Figure 5:**
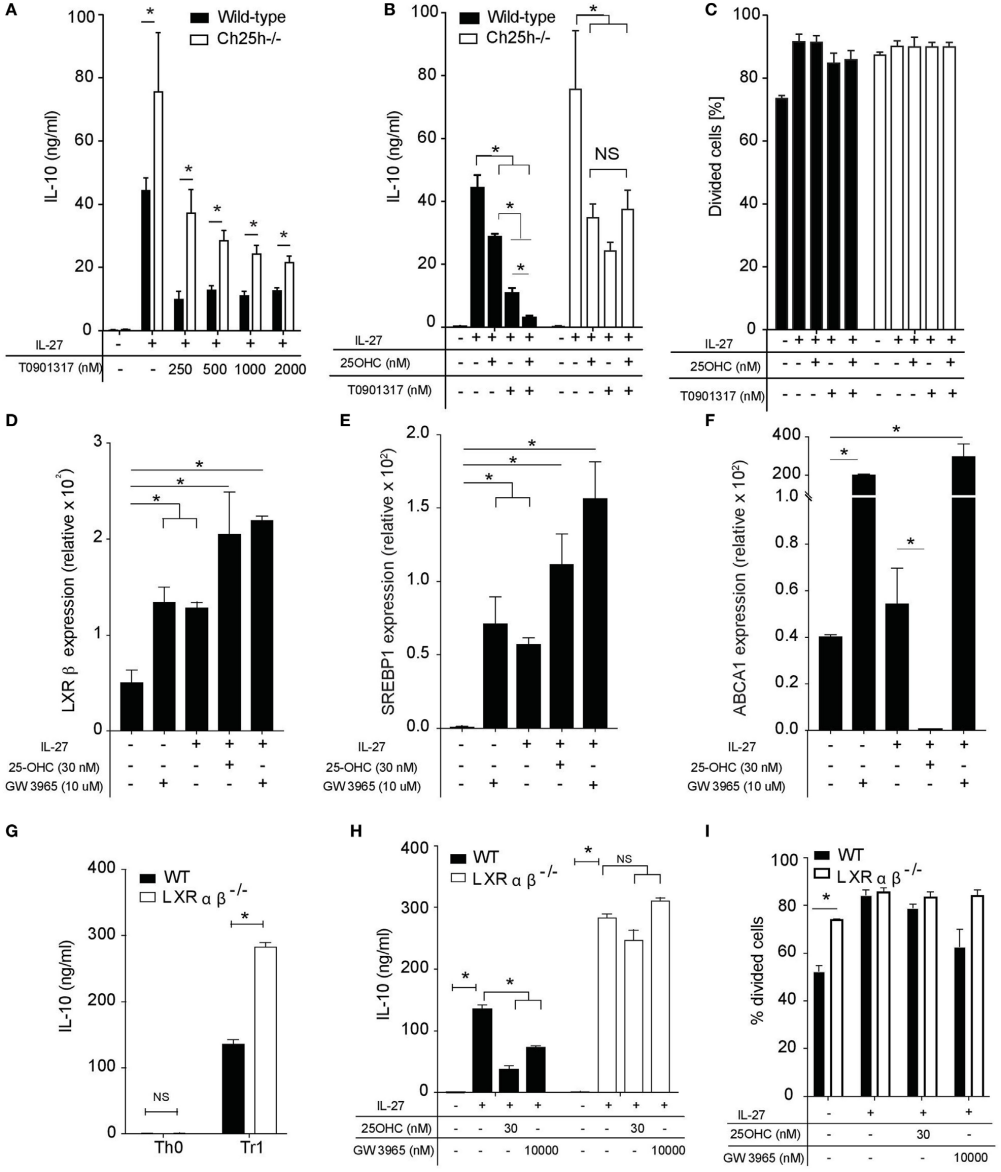
25-OHC negatively IL-10 production *via* liver X receptor (LXR) signaling. Wild-type or Ch25h^−/−^ naive CD4^+^ T cells were differentiated with IL-27 in the presence of indicated concentration of LXR agonist T0901317 alone or 25-OHC. **(A,B)** IL-10 cytokine production was measured by ELISA analysis. **(C)** Percentage of divided cells as assessed by CSFE labeling. Data shown are representative of one of three independent experiments with similar results. Error bars represent SD of triplicates in the same experiment (*p < 0.05). **(D–F)** Total mRNA was isolated after 18 h of culture and analyses by quantitative real-time PCR. Results represent **(D)** LXRβ, **(E)** sterol regulatory element binding protein (SREBP1), and **(F)** ATP-binding cassette transporter A1 (ABCA1) mRNA expression levels relative to β-actin (2^−ΔCT^ × 100,000). Error bars represent the SD of the mean of three independent experiments (*p < 0.05). **(G,H)** IL-10 secretion in 48 h culture supernatants was determined by ELISA. **(I)** Percentage of divided cells as assessed by CSFE labeling. Data are shown from one of two independent experiments with similar results. Error bars represent the SD of triplicates in the same experiment (*p < 0.05).

25-OHC are ligands for the extracellular receptor G-coupled protein receptor Epstein-Barr virus-induced G-protein coupled receptor 2 (EBI2) (32, 33) that is expressed on activated murine and human CD4^+^ T cells (3, 4) and for intracellular receptors. We first assessed whether EBI2 receptor was involved in IL-10 inhibition. Neither IL-10 nor IFN-γ inhibitions by 25-OHC were mediated by EBI2 (data not shown). In addition to EBI2 binding, oxysterols activate transcription factors intracellularly. In this line, 7β,27-dihydroxycholesterol is a potent and selective activator for the transcription factor RORγt, a main transcription factor of T_H_17 cells (13). Furthermore, LXRs are established targets of 22-OHC and 25-OHC (34, 35). We, therefore, tested whether LXR activation would reproduce 25-OHC effects. We first observed that the LXR agonist T0901317 decreased IL-10 expression induced by IL-27 in a dose-dependent manner in wild-type and Ch25h^−/−^ T_R_1 cells (Figure 5A). This LXR agonist was more potent in inhibiting IL-10 secretion in wild-type compared to Ch25h^−/−^ T_R_1 cells (Figure 5A), suggesting a putative additive effect of LXR agonist in the presence of 25-OHC. In this line, sole addition of T0901317 decreased the secretion of IL-10 in the same range than 25-OHC alone; however, combined treatment with 25-OHC and T0901317 depicted additive effects on dampening IL-10 secretion in wild-type but not in Ch25h^−/−^ T_R_1 cells (Figure 5B). No effects on proliferation were observed at the concentration used in this assay (Figure 5C).

To further investigate the specific role of LXR signaling in T_R_1 cells, we examined the expression pattern of LXR-α and LXR-β in naive CD4^+^ T cells (T_H_0) and in IL-27-differentiated T_R_1 cells in the presence or absence of 25-OHC or LXR agonist GW 3965 used as positive control. LXR-α was not detected in any of the above conditions in accordance with previous reports (36). While LXR-β was upregulated in the presence of IL-27 (T_R_1 cells) compared to T_H_0, the addition of 25-OHC on T_R_1 cells significantly enhanced LXR-β mRNA expression to an extent comparable with that induced by GW3965 (Figure 5D). In contrast, the addition of 25-OHC or GW3965 in the absence of IL-27 had no significant effect on LXR-β mRNA expression (Figure S2A in Supplementary Material). To determine whether 25-OHC influences LXR transcriptional program in T_R_1 cells, we tested if 25-OHC could impact LXR-target gene expression particularly genes involved in *de novo* cholesterol biosynthesis as sterol regulatory element binding protein (SREBP1) and in cholesterol efflux as ATP-binding cassette transporter A1 (ABCA1). SREBP1 mRNA expression levels were significantly upregulated compared to T_H_0 when 25-OHC or GW3965 were added together with IL-27 (Figure 5E). Treatment of T_R_1 cells with 25-OHC resulted in a significant downregulation of ABCA1 mRNA expression whereas addition of GW3965 led to a robust induction of this gene (Figure 5F). In contrast, addition of 25-OHC without IL-27 had no significant effect on SREBP1 or ABCA1 mRNA expression (Figures S2B,C in Supplementary Material). These results suggest that LXR is more active in T_R_1 compared to T_H_0 cells.

We then investigated whether the inhibitory effect of 25-OHC on T_R_1 differentiation and IL-10 production was dependent on LXR signaling. We took advantage of cells deficient for LXRαβ. We observed that T_R_1 cells differentiated in the absence of LXRαβ displayed a significantly higher secretion of IL-10 (Figure 5G). 25-OHC was significantly more potent in inhibiting IL-10 production in the presence of LXR receptor similarly to the LXR agonist GW3965 (Figure 5H). To assess the implication of T cell proliferation, WT and LXRαβ^−/−^ naive CD4^+^ T cells were labeled with CFSE and activated with IL-27 in the presence or not of 25-OHC. The percentage of divided cells in response to TCR stimulus alone (anti-CD3, anti-CD28 activation) was significantly greater in LXRαβ^−/−^ compared to WT T_H_0 cells (Figure 5I) in agreement with previous studies (36). However, WT and LXRαβ^−/−^ T_R_1 cells underwent the same percentage of proliferation independently of 25-OHC addition. Altogether, these results indicate that 25-OHC regulates IL-10 production and that LXR signaling mediates, at least partially, the inhibitory effect of 25-OHC on T_R_1 cell polarization.

## Discussion

**Figure 6:**
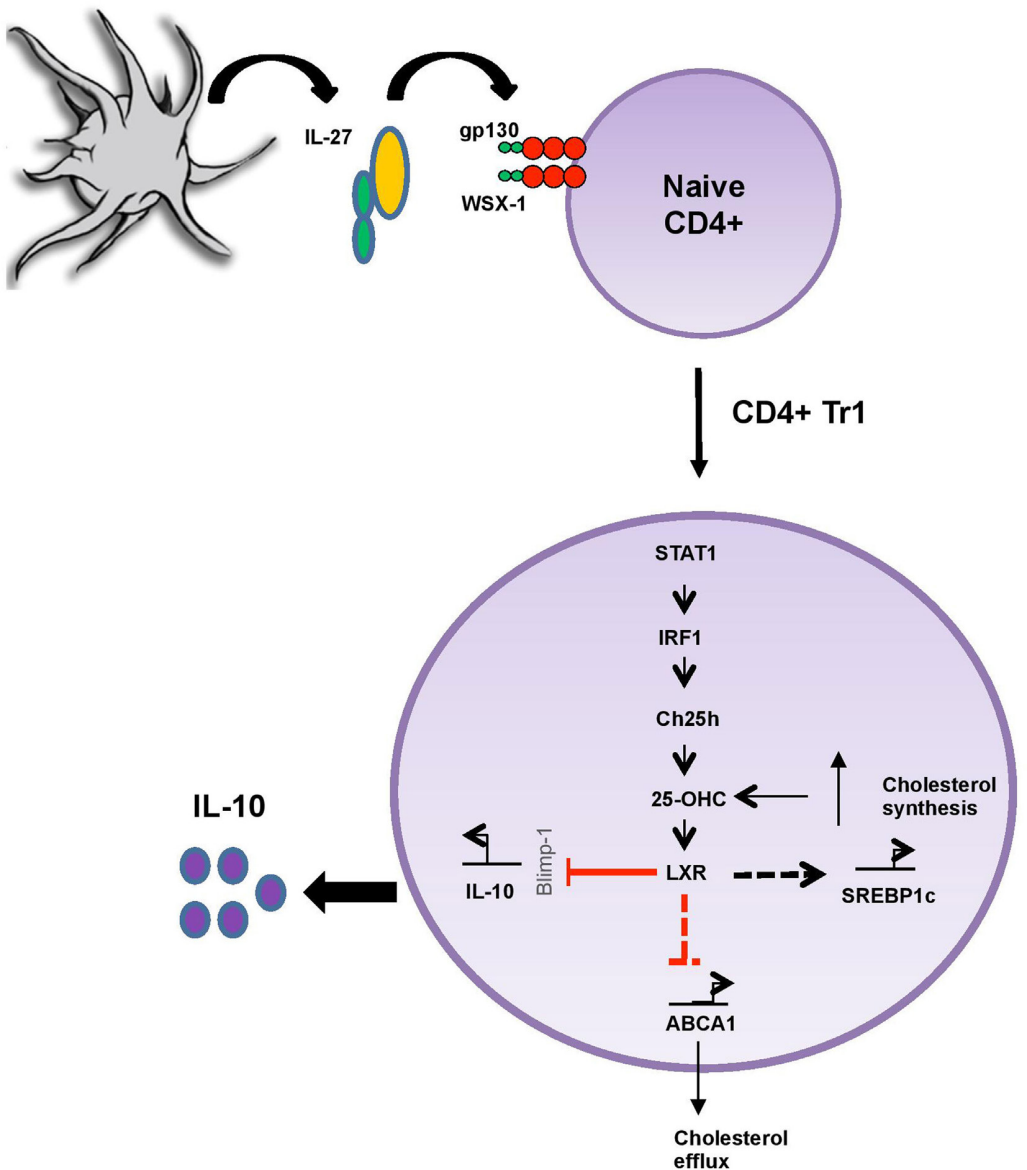
25-OHC acts as an important pro-inflammatory intrinsic regulator of liver X receptor (LXR) during adaptive immune response. IL-27/IL-27R (gp130/WSX-1) signaling promotes 25-OHC production in a STAT1 and interferon regulatory factor 1 (IRF1)-dependent manner through the induction of the enzyme cholesterol 25-hydroxylase (Ch25h). 25-OHC then activates LXR and limits IL-10 secretion, possibly by decreasing the expression levels of the transcription factor Blimp1, known to induce IL-10. In addition, 25-OHC regulates cholesterol homeostasis by increasing sterol regulatory element binding protein (SREBP1) expression involved in its biosynthesis while simultaneously decreasing its efflux through the inhibition ATP-binding cassette transporter A1 (ABCA1) expression. In summary 25-OHC/LXR leads to both sustained inhibition of IL-10-immunosuppressive response and to the accumulation of cholesterol within T_R_1 cells.

Oxysterols have been ascribed functions in modulating the immune response. However, their pro-inflammatory and/or anti-inflammatory contributions remain debated and scarcely studied during adaptive immune response. Here, we propose that 25-OHC dampens the secretion of the major anti-inflammatory cytokine IL-10 induced by IL-27 and thus assigns this oxysterol with a pro-inflammatory role during adaptive immune responses (Figure 6). Our findings are in line with publications assigning 25-OHC with both a pro-inflammatory function and an amplificatory inflammatory signal (1, 37–39).

We observed that Ch25h mRNA expression and 25-OHC levels are strongly induced by IL-27. Both Stat1 and Stat3 are phosphorylated upon IL-27 signaling, leading to transactivation of IL-10 (23, 40). While Stat3 is important for the expression of the transcription factors c-maf and Ahr, Stat1 induces the expression of the transcription factors T-bet, IRF1, and Blimp1 (17, 24). Interestingly, Stat1 (1) and IRF1 (25) can drive Ch25h induction in macrophages during viral infection. IRF1, initially identified as a T_H_1 cell-specific transcription factor, was further implicated in the biology of other T cell subsets such as T_H_9 cells (17, 41). In our study, we observed that Ch25h expression is dependent on the transcription factors Stat1 and IRF1 (Figure 6) but not T-bet. Those results suggest similarities in signaling pathways between innate (in particular macrophages) and adaptive immune responses in inducing cholesterol and oxysterol metabolism. Moreover, we showed that 25-OHC downregulated Blimp1 expression that is induced by IL-12 and IL-27 and promotes IL-10 production in T cells (42). Our observations suggest that 25-OHC suppress IL-10 secretion from T_R_1 cells by antagonizing Blimp1 expression.

Cholesterol is converted by the enzyme Ch25h to 25-OHC, which can be further metabolized into 7α,25-OHC in the presence of the cytochrome Cyp7b1. This latter cytochrome is abundant in the liver where it mediates bile acid synthesis. By contrast, Ch25h is poorly expressed in healthy liver, leading to an early suggestion that 25-OHC might generate other biological processes than bile acid production. We observed that IL-27 induced the expression of Ch25h but not Cyp7b1, leading to the production of 25-OHC but not of 7α,25-OHC. This is strengthened by the fact that the effect of 25-OHC on T cells is not dependent on EBI2 expression, a G-protein coupled receptor that binds 7α,25-OHC and 25-OHC with high and modest affinity, respectively.

25-OHC controls transcriptional activities intracellularly and binds to several transcription factors including RORγt (13, 43, 44) and LXRs (34, 35). LXRα and LXRβ have been implicated in cholesterol and fatty acid homeostasis *via* regulation of reverse cholesterol transport and *de novo* fatty acid synthesis. In addition, they regulate inflammatory gene expression and immune cell proliferation. Mechanistically, their effects are attributed to the inhibition of nuclear factor kappa-B and Stat1-mediated signaling pathways (45). LXRs biology has been studied in macrophages principally in atherosclerosis development (46) and more recently in T cells during autoimmune disorders, including experimental autoimmune encephalomyelitis and arthritis models (47–49). In agreement with previous studies, we here show that LXRβ is expressed in activated T cells (36). Interestingly, we observed that LXRβ mRNA is upregulated in IL-27-differentiated Tr1 cells and that addition of exogenous 25-OHC is able to significantly increase LXRβ expression in these cells but not in activated Th0 cells. Moreover, the measurement of 25-OHC within the cellular compartment of T_R_1 cells emphasizes our hypothesis that 25-OHC acts as an intrinsic transcriptional regulator of LXR. In T_R_1 cells 25-OHC induced the expression of SREBP1 and repressed the expression of ABCA1 (Figure 6) in contrary to what is known in macrophages in which oxysterols inhibit the maturation of SREBP *via* an LXR-independent pathway and induce the transcription of ABCA1 under conditions of cholesterol excess (35). Previous studies on lymphocytes have shown that genes encoding sterol transporters or fatty acids synthesis like ABCA1, ABCG1, or SREBP1 are strongly stimulated upon the addition of synthetic LXR agonist like GW3965 or T0901317 (36, 50). However, the influence of lipid metabolism on CD4^+^ T lymphocyte function is still poorly understood.

Liver X receptors have been ascribed anti-inflammatory functions. They have been proposed to negatively regulate macrophages inflammatory gene expression (51) and to inhibit T_H_17 cell generation and thus to mediate anti-inflammatory signals during adaptive immune responses (47). Moreover, LXRs agonist could affect different subsets of T cells including T_H_1, T_H_2, and iTreg by limiting T cell proliferation (50). We observed that low concentration of 25-OHC limits the anti-inflammatory response induced by IL-27 in T_R_1 cells *via* LXR signaling. The production of 25-OHC by T_R_1 cells is in agreement with the existence of an autocrine and paracrine 25-OHC/LXR amplification loop, inhibiting both T_R_1 polarization and cholesterol efflux while enhancing cholesterol production by T_R_1 cells (Figure 6). Finally, intracellular cholesterol accumulation has been shown to promote inflammation in innate immunity (15). Oxysterols can thus be considered as fine tuners of inflammation and cholesterol homeostasis during adaptive immune responses.

In humans, T_R_1 cells were first described in severe combined immunodeficient patients who had developed long-term tolerance to stem cell allografts, suggesting that these cells might naturally regulate immune responses in humans (52). However, human Treg play a deleterious role in cancer as they mediate suppression of antitumor responses and also interfere with immunotherapies. Tumor-associated human T_R_1 have been shown to be pro-tumorigenic, as they mediate immune suppression (53). Harnessing T_R_1 cells by modulating cholesterol pathways might open new tools in immunotherapy.

In conclusion, as one of the suppressive T cell subsets, T_R_1 cells have been described to regulate inflammation, graft-versus-host disease and autoimmunity by producing IL-10. However, excess of anti-inflammatory response may lead to uncontrolled infections or tumor development. The results presented in this study show that IL-27, a main inducer of T_R_1 cells, induces oxysterols to regulate the strength of the anti-inflammatory response. Taken together, our study identifies Ch25h and its biosynthetic product 25-OHC as negative regulators induced by IL-27 to maintain immune homeostasis *via* LXR signaling. Here, the induction of oxysterols would limit the induction of regulatory T cells to prevent excessive immune regulation that might favor the emergence of viral infections or cancers.

## Ethics Statement

All procedures and methods were approved by the Cantonal Veterinary Services (SCAV, autorisations VD 3025 and GE 1914).

## Author Contributions

SV, FC, DD, and AC performed experiments and analyzed results; CP designed the research; LA provided IRF1^−/−^ mice and scientific advises; J-ML provided LXRαβ KO mice and scientific advises; JZ and IC performed mass spectrometry analysis and provided scientific advises; and SV and CP elaborated the figures and wrote the paper.

## Conflict of Interest Statement

The authors have no conflict-of-interest to declare. IC and JZ are employees of Novartis Pharma AG and hold stock and stock options in their company. The authors have no additional financial interests to declare.

## References

[1] Blanc M Hsieh WY Robertson KA Kropp KA Forster T Shui G The transcription factor STAT-1 couples macrophage synthesis of 25-hydroxycholesterol to the interferon antiviral response. Immunity (2013) 38:106–18. 10.1016/j.immuni.2012.11.004 23273843 PMC3556782

[2] Yi T Wang X Kelly LM An J Xu Y Sailer AW Oxysterol gradient generation by lymphoid stromal cells guides activated B cell movement during humoral responses. Immunity (2012) 37:535–48. 10.1016/j.immuni.2012.06.015 22999953 PMC3465460

[3] Chalmin F Rochemont V Lippens C Clottu A Sailer AW Merkler D Oxysterols regulate encephalitogenic CD4(+) T cell trafficking during central nervous system autoimmunity. J Autoimmun (2015) 56:45–55. 10.1016/j.jaut.2014.10.001 25456971

[4] Clottu AS Mathias A Sailer AW Schluep M Seebach JD Du Pasquier R EBI2 expression and function: robust in memory lymphocytes and increased by natalizumab in multiple sclerosis. Cell Rep (2017) 18:213–24. 10.1016/j.celrep.2016.12.006 28052250

[5] Reboldi A Dang EV McDonald JG Liang G Russell DW Cyster JG. Inflammation. 25-Hydroxycholesterol suppresses interleukin-1-driven inflammation downstream of type I interferon. Science (2014) 345:679–84. 10.1126/science.1254790 25104388 PMC4289637

[6] Fitzgerald DC Ciric B Touil T Harle H Grammatikopolou J Das Sarma J Suppressive effect of IL-27 on encephalitogenic Th17 cells and the effector phase of experimental autoimmune encephalomyelitis. J Immunol (2007) 179:3268–75. 10.4049/jimmunol.179.5.3268 17709543

[7] Murugaiyan G Mittal A Weiner HL. Identification of an IL-27/osteopontin axis in dendritic cells and its modulation by IFN-gamma limits IL-17-mediated autoimmune inflammation. Proc Natl Acad Sci U S A (2010) 107:11495–500. 10.1073/pnas.1002099107 20534530 PMC2895126

[8] Fitzgerald DC Rostami A. Therapeutic potential of IL-27 in multiple sclerosis? Expert Opin Biol Ther (2009) 9:149–60. 10.1517/14712590802646936 19236245

[9] Stumhofer JS Laurence A Wilson EH Huang E Tato CM Johnson LM Interleukin 27 negatively regulates the development of interleukin 17-producing T helper cells during chronic inflammation of the central nervous system. Nat Immunol (2006) 7:937–45. 10.1038/ni1376 16906166

[10] Batten M Li J Yi S Kljavin NM Danilenko DM Lucas S Interleukin 27 limits autoimmune encephalomyelitis by suppressing the development of interleukin 17-producing T cells. Nat Immunol (2006) 7:929–36. 10.1038/ni1375 16906167

[11] Villarino A Hibbert L Lieberman L Wilson E Mak T Yoshida H The IL-27R (WSX-1) is required to suppress T cell hyperactivity during infection. Immunity (2003) 19:645–55. 10.1016/S1074-7613(03)00300-5 14614852

[12] Awasthi A Carrier Y Peron JP Bettelli E Kamanaka M Flavell RA A dominant function for interleukin 27 in generating interleukin 10-producing anti-inflammatory T cells. Nat Immunol (2007) 8:1380–9. 10.1038/ni1541 17994022

[13] Soroosh P Wu J Xue X Song J Sutton SW Sablad M Oxysterols are agonist ligands of RORgammat and drive Th17 cell differentiation. Proc Natl Acad Sci U S A (2014) 111:12163–8. 10.1073/pnas.1322807111 25092323 PMC4143045

[14] Heinemann C Heink S Petermann F Vasanthakumar A Rothhammer V Doorduijn E IL-27 and IL-12 oppose pro-inflammatory IL-23 in CD4+ T cells by inducing Blimp1. Nat Commun (2014) 5:3770. 10.1038/ncomms4770 24796719

[15] Ito A Hong C Oka K Salazar JV Diehl C Witztum JL Cholesterol accumulation in CD11c+ immune cells is a causal and targetable factor in autoimmune disease. Immunity (2016) 45:1311–26. 10.1016/j.immuni.2016.11.008 28002731 PMC5181791

[16] Wallner B Leitner NR Vielnascher RM Kernbauer E Kolbe T Karaghiosoff M Generation of mice with a conditional Stat1 null allele. Transgenic Res (2012) 21:217–24. 10.1007/s11248-011-9519-5 21553074

[17] Vegran F Berger H Boidot R Mignot G Bruchard M Dosset M The transcription factor IRF1 dictates the IL-21-dependent anticancer functions of TH9 cells. Nat Immunol (2014) 15:758–66. 10.1038/ni.2925 24973819

[18] Repa JJ Turley SD Lobaccaro JA Medina J Li L Lustig K Regulation of absorption and ABC1-mediated efflux of cholesterol by RXR heterodimers. Science (2000) 289:1524–9. 10.1126/science.289.5484.1524 10968783

[19] Karuna R Christen I Sailer AW Bitsch F Zhang J. Detection of dihydroxycholesterols in human plasma using HPLC-ESI-MS/MS. Steroids (2015) 99:131–8. 10.1016/j.steroids.2015.02.002 25683891

[20] Smith LL. Cholesterol autoxidation 1981-1986. Chem Phys Lipids (1987) 44:87–125. 10.1016/0009-3084(87)90046-6 3311423

[21] Lund E Bjorkhem I Furster C Wikvall K. 24-, 25- and 27-hydroxylation of cholesterol by a purified preparation of 27-hydroxylase from pig liver. Biochim Biophys Acta (1993) 1166:177–82. 10.1016/0005-2760(93)90094-P 8443234

[22] Pot C Apetoh L Kuchroo VK. Type 1 regulatory T cells (Tr1) in autoimmunity. Semin Immunol (2011) 23:202–8. 10.1016/j.smim.2011.07.005 21840222 PMC3178065

[23] Peters A Fowler KD Chalmin F Merkler D Kuchroo VK Pot C. IL-27 induces Th17 differentiation in the absence of STAT1 signaling. J Immunol (2015) 195:4144–53. 10.4049/jimmunol.1302246 26408664 PMC4610870

[24] Amadi-Obi A Yu CR Dambuza I Kim SH Marrero B Egwuagu CE. Interleukin 27 induces the expression of complement factor H (CFH) in the retina. PLoS One (2012) 7:e45801. 10.1371/journal.pone.0045801 23029250 PMC3447806

[25] Mboko WP Mounce BC Emmer J Darrah E Patel SB Tarakanova VL. Interferon regulatory factor 1 restricts gammaherpesvirus replication in primary immune cells. J Virol (2014) 88:6993–7004. 10.1128/JVI.00638-14 24719409 PMC4054362

[26] de Weille J Fabre C Bakalara N Oxysterols in cancer cell proliferation and death. Biochem Pharmacol (2013) 86:154–60. 10.1016/j.bcp.2013.02.029 23500545

[27] Kamanaka M Kim ST Wan YY Sutterwala FS Lara-Tejero M Galan JE Expression of interleukin-10 in intestinal lymphocytes detected by an interleukin-10 reporter knockin tiger mouse. Immunity (2006) 25:941–52. 10.1016/j.immuni.2006.09.013 17137799

[28] Apetoh L Quintana FJ Pot C Joller N Xiao S Kumar D The aryl hydrocarbon receptor interacts with c-Maf to promote the differentiation of type 1 regulatory T cells induced by IL-27. Nat Immunol (2010) 11:854–61. 10.1038/ni.1912 20676095 PMC2940320

[29] Xu J Yang Y Qiu G Lal G Wu Z Levy DE c-Maf regulates IL-10 expression during Th17 polarization. J Immunol (2009) 182:6226–36. 10.4049/jimmunol.0900123 19414776 PMC2834209

[30] Maynard CL Harrington LE Janowski KM Oliver JR Zindl CL Rudensky AY Regulatory T cells expressing interleukin 10 develop from Foxp3+ and Foxp3- precursor cells in the absence of interleukin 10. Nat Immunol (2007) 8:931–41. 10.1038/ni1504 17694059

[31] Pot C Jin H Awasthi A Liu SM Lai CY Madan R Cutting edge: IL-27 induces the transcription factor c-Maf, cytokine IL-21, and the costimulatory receptor ICOS that coordinately act together to promote differentiation of IL-10-producing Tr1 cells. J Immunol (2009) 183:797–801. 10.4049/jimmunol.0901233 19570826 PMC2768608

[32] Hannedouche S Zhang J Yi T Shen W Nguyen D Pereira JP Oxysterols direct immune cell migration via EBI2. Nature (2011) 475:524–7. 10.1038/nature10280 21796212 PMC4297623

[33] Liu C Yang XV Wu J Kuei C Mani NS Zhang L Oxysterols direct B-cell migration through EBI2. Nature (2011) 475:519–23. 10.1038/nature10226 21796211

[34] Janowski BA Willy PJ Devi TR Falck JR Mangelsdorf DJ An oxysterol signalling pathway mediated by the nuclear receptor LXR alpha. Nature (1996) 383:728–31. 10.1038/383728a0 8878485

[35] Spann NJ Glass CK. Sterols and oxysterols in immune cell function. Nat Immunol (2013) 14:893–900. 10.1038/ni.2681 23959186

[36] Bensinger SJ Bradley MN Joseph SB Zelcer N Janssen EM Hausner MA LXR signaling couples sterol metabolism to proliferation in the acquired immune response. Cell (2008) 134:97–111. 10.1016/j.cell.2008.04.052 18614014 PMC2626438

[37] Gold ES Diercks AH Podolsky I Podyminogin RL Askovich PS Treuting PM 25-Hydroxycholesterol acts as an amplifier of inflammatory signaling. Proc Natl Acad Sci U S A (2014) 111:10666–71. 10.1073/pnas.1404271111 24994901 PMC4115544

[38] Gold ES Ramsey SA Sartain MJ Selinummi J Podolsky I Rodriguez DJ ATF3 protects against atherosclerosis by suppressing 25-hydroxycholesterol-induced lipid body formation. J Exp Med (2012) 209:807–17. 10.1084/jem.20111202 22473958 PMC3328364

[39] Liu SY Aliyari R Chikere K Li G Marsden MD Smith JK Interferon-inducible cholesterol-25-hydroxylase broadly inhibits viral entry by production of 25-hydroxycholesterol. Immunity (2013) 38:92–105. 10.1016/j.immuni.2012.11.005 23273844 PMC3698975

[40] Stumhofer JS Silver JS Laurence A Porrett PM Harris TH Turka LA Interleukins 27 and 6 induce STAT3-mediated T cell production of interleukin 10. Nat Immunol (2007) 8:1363–71. 10.1038/ni1537 17994025

[41] Taki S Sato T Ogasawara K Fukuda T Sato M Hida S Multistage regulation of Th1-type immune responses by the transcription factor IRF-1. Immunity (1997) 6:673–9. 10.1016/S1074-7613(00)80443-4 9208840

[42] Neumann C Heinrich F Neumann K Junghans V Mashreghi MF Ahlers J Role of Blimp-1 in programing Th effector cells into IL-10 producers. J Exp Med (2014) 211:1807–19. 10.1084/jem.20131548 25073792 PMC4144744

[43] Jin L Martynowski D Zheng S Wada T Xie W Li Y. Structural basis for hydroxycholesterols as natural ligands of orphan nuclear receptor RORgamma. Mol Endocrinol (2010) 24:923–9. 10.1210/me.2009-0507 20203100 PMC2870936

[44] Huh JR Leung MW Huang P Ryan DA Krout MR Malapaka RR Digoxin and its derivatives suppress TH17 cell differentiation by antagonizing RORgammat activity. Nature (2011) 472:486–90. 10.1038/nature09978 21441909 PMC3172133

[45] Ghisletti S Huang W Jepsen K Benner C Hardiman G Rosenfeld MG Cooperative NCoR/SMRT interactions establish a corepressor-based strategy for integration of inflammatory and anti-inflammatory signaling pathways. Genes Dev (2009) 23:681–93. 10.1101/gad.1773109 19299558 PMC2661610

[46] Calkin AC Tontonoz P Liver X receptor signaling pathways and atherosclerosis. Arterioscler Thromb Vasc Biol (2010) 30:1513–8. 10.1161/ATVBAHA.109.191197 20631351 PMC2919217

[47] Cui G Qin X Wu L Zhang Y Sheng X Yu Q Liver X receptor (LXR) mediates negative regulation of mouse and human Th17 differentiation. J Clin Invest (2011) 121:658–70. 10.1172/JCI42974 21266776 PMC3026720

[48] Asquith DL Miller AM Hueber AJ McKinnon HJ Sattar N Graham GJ Liver X receptor agonism promotes articular inflammation in murine collagen-induced arthritis. Arthritis Rheum (2009) 60:2655–65. 10.1002/art.24717 19714646

[49] Hindinger C Hinton DR Kirwin SJ Atkinson RD Burnett ME Bergmann CC Liver X receptor activation decreases the severity of experimental autoimmune encephalomyelitis. J Neurosci Res (2006) 84:1225–34. 10.1002/jnr.21038 16955483

[50] Solt LA Kamenecka TM Burris TP. LXR-mediated inhibition of CD4+ T helper cells. PLoS One (2012) 7:e46615. 10.1371/journal.pone.0046615 23029557 PMC3460920

[51] Joseph SB Castrillo A Laffitte BA Mangelsdorf DJ Tontonoz P. Reciprocal regulation of inflammation and lipid metabolism by liver X receptors. Nat Med (2003) 9:213–9. 10.1038/nm820 12524534

[52] Groux H Bigler M de Vries JE Roncarolo MG. Interleukin-10 induces a long-term antigen-specific anergic state in human CD4+ T cells. J Exp Med (1996) 184:19–29. 10.1084/jem.184.1.19 8691133 PMC2192687

[53] Bergmann C Strauss L Zeidler R Lang S Whiteside TL. Expansion and characteristics of human T regulatory type 1 cells in co-cultures simulating tumor microenvironment. Cancer Immunol Immunother (2007) 56:1429–42. 10.1007/s00262-007-0280-9 17265021 PMC11031003

