# Supplementary material for: IL-27-Induced Type 1 Regulatory T-Cells Produce Oxysterols that Constrain IL-10 Production
Source: Front Immunol. 2017 Sep 25;8:1184. doi: 10.3389/fimmu.2017.01184 (PMC5622150; doi:10.3389/fimmu.2017.01184)
Supplement: Supplementary file 1 [file Image_1.PDF]

Figure S1

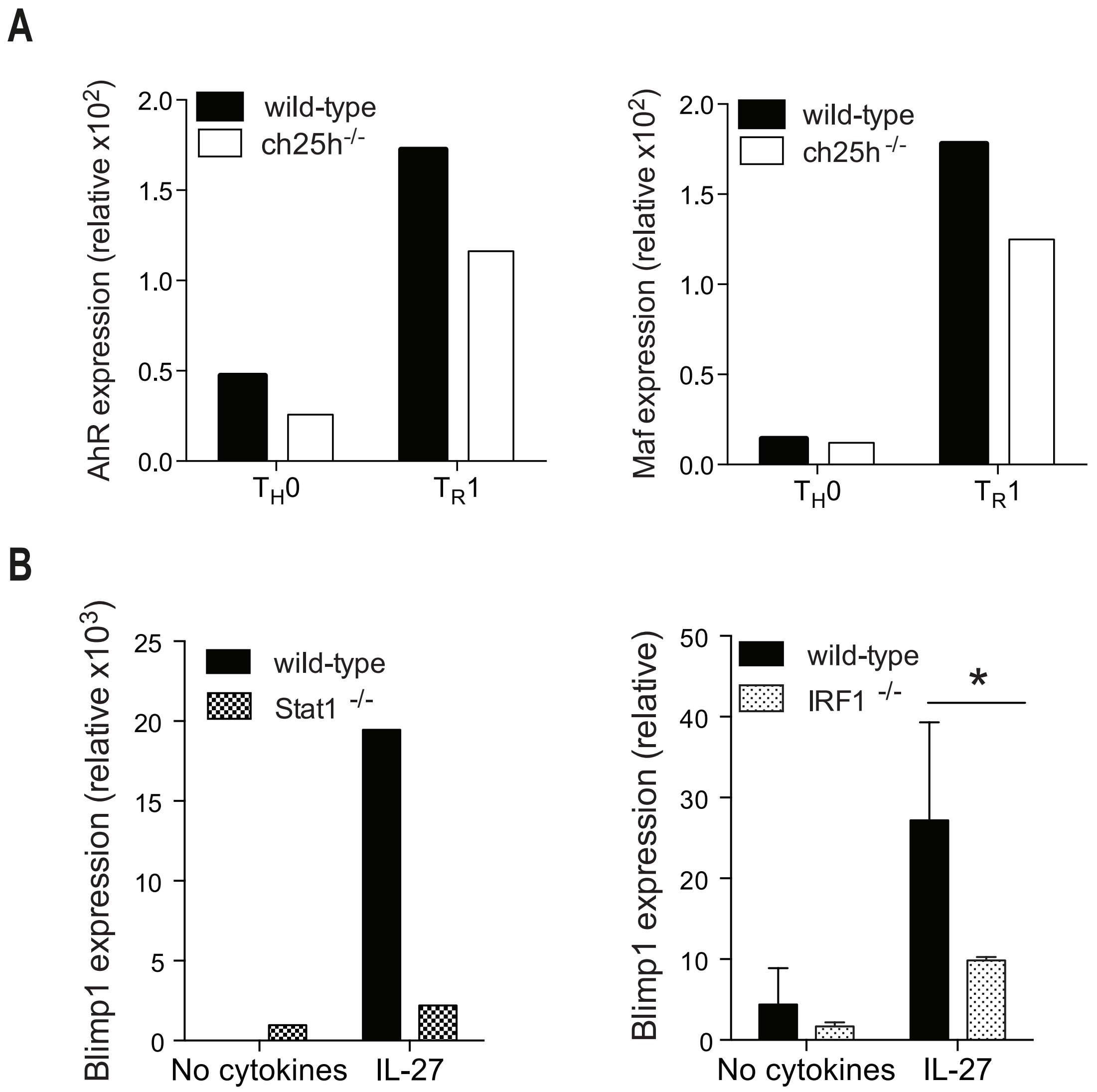

A
AhR expression (relative x10^2)
2.0
1.5
1.0
0.5
0.0
wild-type
ch25h-/-
TH0
TR1
Maf expression (relative x10^2)
2.0
1.5
1.0
0.5
0.0
wild-type
ch25h -/-
TH0
TR1
B
Blimp1 expression (relative x10^3)
25
20
15
10
5
0
wild-type
Stat1 -/-
No cytokines
IL-27
Blimp1 expression (relative)
50
40
30
20
10
0
wild-type
IRF1 -/-
*
No cytokines
IL-27

Figure S1. Ahr and c-maf expressions are not regulated by Ch25h while Blimp1 is dependent of Stat1 and IRF1 signaling.

(A) Naive T cells from wild-type and Ch25h$^{-/-}$ were differentiated with IL-27. After 24 hours, RNA was isolated from the CD4^{+} T cells in culture and subjected to quantitative real-time PCR (RT-PCR) relative to the expression of mRNA encoding β-actin to examine Ahr and c-maf expression. **(B)** Blimp1 expression was assessed by RT-PCR relative to β-actin from RNA was isolated after 48 hours of culture from T_R1 cells obtained from wild-type versus Stat1$^{-/-}$ or IRF1$^{-/-}$ mice

Figure S2

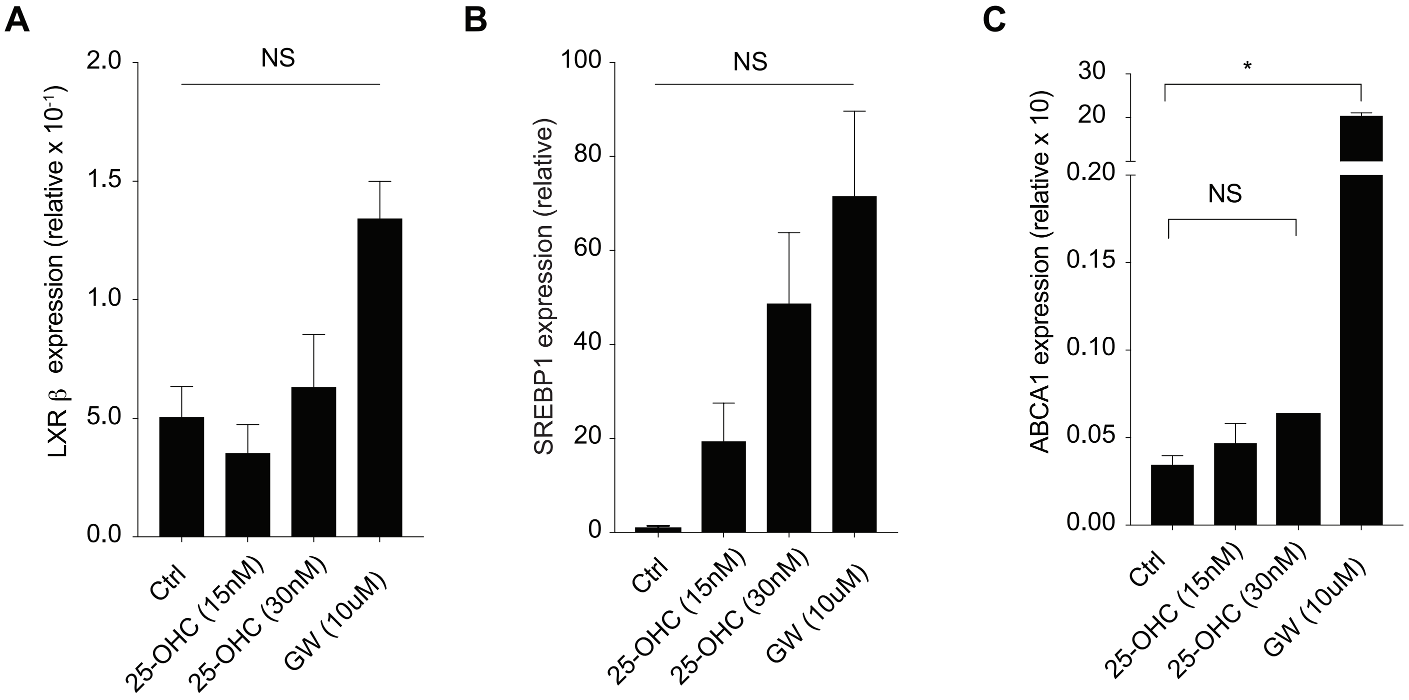

A
B
C
LXR β expression (relative x 10⁻¹)
SREBP1 expression (relative)
ABCA1 expression (relative x 10)
NS
NS
NS
*
2.0
1.5
1.0
5.0
0.0
100
80
60
40
20
0
30
20
0.20
0.15
0.10
0.05
0.00
Ctrl
25-OHC (15nM)
25-OHC (30nM)
GW (10uM)

Figure S2. 25-OHC alone does not induce LXR signaling in Ctrl cells (activated T_H0 cells).

Wild-type naive $CD4^+$ T cells were activated in the presence of indicated concentration of 25-OHC or LXR agonist alone as indicated. Total mRNA was isolated and analyses by quantitative RT-PCR performed. Results represent **(A)** LXRβ, **(B)** SREBP1, and **(C)** ABCA1 mRNA expression levels relative to β-actin.
